# Institutional Decoupling in China’s Blood Donation Reform: Bridging the Gap Between Policy Intentions and Implementation Realities

**DOI:** 10.34172/ijhpm.9536

**Published:** 2026-02-02

**Authors:** Renqi Luo, Jingrong Lu

**Affiliations:** College of Humanities & Social Sciences, Huazhong Agricultural University, Wuhan, China.

## Introduction

 Healthcare quality depends fundamentally on the alignment between policy intentions and clinical realities. When well-intentioned reforms encounter complex operational realities and structural limitations, a critical divide emerges, a phenomenon widely referred to as the theory-policy-practice gap.^1^ China’s blood donation system offers a paradigmatic case of this phenomenon.

 Since 2018, mutual blood donation has been officially restricted to align with World Health Organization (WHO) standards. However, hospitals, compelled by blood shortages, continue requiring families to provide blood volumes equivalent to surgical needs before major surgeries can proceed. This creates a state of “institutional decoupling,” where official rules and operational reality diverge. As a populous middle-income country rapidly transitioning from an incentive-based to a purely voluntary system, China’s experience highlights the friction between international best practices and local resource constraints. While national statistics indicate progress, this growth has struggled to keep pace with the surging clinical demand in major medical centers.

 This disconnect creates fundamental deficiencies in equity. The result is a system where official rules and operational reality exist in parallel worlds, each undermining the other, while patients bear the burden of navigating both. Policy-makers must design policies that account for implementation realities, resource constraints, institutional capacity, and social contexts. Understanding why well-intentioned policies fail in practice offers actionable insights for decision-makers and health system managers navigating complex trade-offs between ideal standards and achievable reforms.

## When Policy and Practice Diverge

 China’s transition from paid to voluntary blood donation began with the 1998 Blood Donation Law, which only permitted mutual blood donation as an emergency mechanism.^2^ Under this arrangement, patients’ relatives or friends could donate blood specifically for their use when blood banks faced shortages. By 2016, however, this practice had grown alarmingly, reaching 35.6% of donations in some provinces. When mutual blood donation exceeds 5% of total donations, the WHO warns of risks including illegal blood trade.^3^ Recognizing these concerns, China’s National Health Commission eliminated mutual blood donation nationwide in 2018, except in remote areas.^4^

 The prohibition appeared sound from a safety perspective. International best practices consistently demonstrate that voluntary, non-remunerated blood donation systems provide the safest blood supply while minimizing commercial exploitation.^5^ Yet within four years, the consequences of the hasty transition became clear. From 2022 onwards, blood centers in multiple regions across China issued critical low-inventory alerts, disrupting the previously maintained supply balance.^6^ By 2024, donation volumes had declined significantly, with China achieving only 11.4 blood donors per 1000 people.^7^ This systematic shortfall forced hospitals back to requiring mutual blood donation.

 This gap between what policies mandate and what actually happens in clinical settings represents more than administrative inconsistency. Research on healthcare implementation demonstrates that such disconnects fundamentally compromise care delivery, creating unpredictable, inequitable systems where patients cannot rely on official processes to meet their needs.^8^ The implications extend beyond blood safety to encompass access, equity, and the broader integrity of healthcare delivery systems. This disconnect fundamentally undermines healthcare quality by creating systematic barriers between clinical needs and available resources, ultimately compromising patient safety through unpredictable care pathways.^9^

## The Burden of Informal Solutions

 The policy-practice gap manifests most acutely for patients traveling from rural areas to urban medical centers.^10^ These families often arrive in major cities for medical treatment with no social networks capable of providing blood donations. When hospitals require families to provide equivalent blood donations before surgery, these families face impossible choices. They must either choose to delay surgery while waiting for family members to gradually travel from their hometowns to big cities for blood donation, or contact strangers for blood donation through blood brokers. The geographic concentration of advanced medical facilities exacerbates this challenge, as patients from across China converge on major urban hospitals while lacking local connections to facilitate donation requirements.

 Our field observations in several major hospitals in Wuhan indicate that official prohibitions have not eliminated the supply-demand gap but have instead fostered complex informal workarounds. For example, in these hospitals, families of patients are often forced to enter shadow markets through intermediaries (commonly known as “blood brokers”), paying approximately 1300 RMB for each 400 mL blood quota to meet hospital requirements. This phenomenon is further corroborated by scholarly analyses, regarding private transactions between blood donors and recipients and the structural mismatch within China’s blood supply chain.^11,12^ While hospitals strictly adhere to the prohibition of mutual donation in procedural terms, the clinical side often tacitly permits these “grey market” contributions due to urgent surgical pressures, processing them through formal channels as “voluntary donations” to maintain institutional compliance. In other words, hospitals addressed unachievable tasks by decoupling “procedural compliance” from “tactical discretion.”^13^

 The problem, then, is not primarily about blood safety from infectious diseases, which remains managed through official screening processes. Rather, it represents a fundamental failure in access and equity that systematically undermines care quality and introduces ethical risks through informal, unregulated care pathways that operate outside established assurance mechanisms. Studies across healthcare systems show that when essential services require informal payments or social connections, care becomes the privilege of those with resources rather than a right based on medical need. Research on healthcare disparities consistently demonstrates that such barriers disproportionately affect rural, low-income, and socially isolated populations.^14^

 Moreover, this unofficial system undermines trust, a cornerstone of effective healthcare delivery.^15^ When patients navigate shadowy networks to access care, when official policies do not reflect operational realities, and when hospitals tacitly permit practices they officially prohibit, the credibility of the entire healthcare system erodes. This erosion of trust itself becomes a systemic issue, affecting patient willingness to seek care and engagement with the health system. The psychological burden on families, already stressed by medical emergencies, intensifies when they must simultaneously navigate informal payment systems and uncertain supply chains.

## Why the Gap Persists

 Understanding why this paradox persists requires examining the complex interplay of structural, environmental, and social factors beyond simple policy design.

 The persistence of the gap is first attributable to profound environmental shocks, notably the “COVID-19 Hangover” and its lasting impact on donor behavior. As widely observed in studies, lockdowns and infection fears significantly disrupted traditional, street-based donor recruitment.^16^ Even with the pandemic’s receding, the recovery of regular donor cohorts has been slower than anticipated, creating persistent volatility in supply that renders the strict prohibition of mutual donation operationally difficult for hospitals facing immediate surgical backlogs.

 Furthermore, geographic structural imbalance compound the challenge. Advanced medical facilities are heavily concentrated in major metropolises like Beijing and Shanghai. Rural areas face insufficient medical resources, and when confronted with complex diseases, choosing hospitals in major cities is a necessary choice. This creates disproportionate demand in urban centers while donor bases remain primarily local. The mismatch between where patients seek care and where donors typically contribute represents a structural design issue that policy prohibition alone cannot resolve. The current policy framework lacks a sufficiently robust national allocation mechanism to redistribute blood products efficiently from surplus regions to deficit centers, forcing patient families to bridge this gap individually.

 This situation is exacerbated by an erosion of public trust in the digital age. Public trust, a cornerstone of any sustainable voluntary system, faces new challenges as social media platforms occasionally amplify negative narratives regarding blood bank transparency or usage. When patients are forced to navigate informal brokers, it validates public skepticism and creates a vicious cycle that further discourages altruistic participation.^17^

 Collectively, these factors lead to “Institutional Decoupling,” a situation where stated rules and actual operations systematically diverge.^18^ International experience demonstrates that successful voluntary blood donation systems require sustained public education, convenient donation opportunities, and community trust in healthcare institutions.^19^ Simply prohibiting one practice without fully establishing adaptive mechanisms creates predictable shortages. Owing to insufficient “policy capacity,” meaning the necessary infrastructure, effective donor incentives, and cultural adaptation, hospitals have been compelled to prioritise patient survival and critical surgical needs pragmatically, rather than strictly adhering to regulatory requirements. In addition, the absence of feedback mechanisms between policy-makers and clinical practitioners perpetuates this dysfunction, as decision-makers receive limited real-time information about implementation challenges.

## Rethinking Evaluation Metrics

 Traditional blood service quality metrics focus primarily on collection volumes and safety screening, but comprehensive evaluation requires examining multiple dimensions. Healthcare systems must consider whether all patient populations can obtain needed blood products regardless of location, connections, or economic status. The reliability of supply becomes crucial as patients and providers must be able to depend on availability when clinically needed. Consistency between official policies and actual practices represents another essential measure, as does the financial, logistical, and emotional burden that patients and families bear to access this essential resource.

 Current practices fail on most of these measures. The policy-practice gap itself indicates severe process inconsistency that compromises quality. The requirement for families to secure blood donations whether through social networks or paid intermediaries represents significant patient burden and access inequity. The underground nature of blood brokerage undermines transparency and accountability. Research on health systems strengthening emphasizes that sustainable improvements cannot be achieved through fragmented interventions. Success requires coherent systems where policies, resources, infrastructure, and practices align to support patient care quality and safety.

 The disconnect also reveals fundamental measurement gaps. Current monitoring systems track donation volumes and safety indicators but fail to capture access barriers, patient burden, or the prevalence of informal workarounds. Without comprehensive data on how the system actually functions for different patient populations, policy-makers cannot design effective interventions or anticipate unintended consequences.

## Building Evidence-Based Solutions

 Resolving this paradox requires addressing root causes through evidence-based approaches rather than policy declarations alone.^20^ The conceptual framework for this decoupling is illustrated in Figure.

**Figure:**
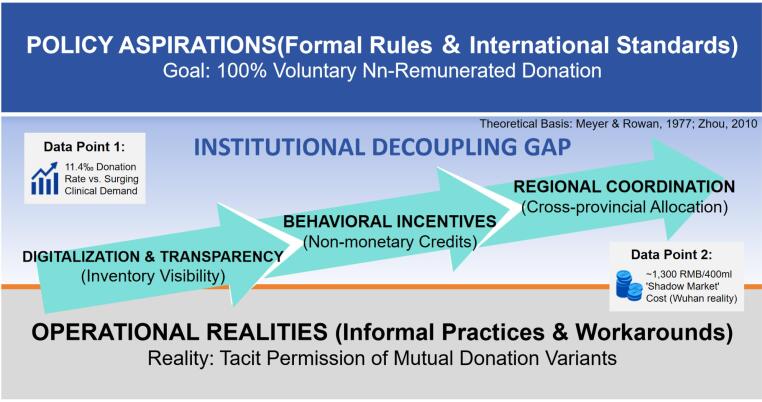
Conceptual Framework of the Decoupling Between Blood Policy and Clinical Practice.

 First, policy-makers must demonstrate genuine commitment to building voluntary donation infrastructure through sustained investment in donor recruitment, public education, and convenient donation opportunities. Authorities must conduct a comprehensive analysis of the reasons behind low resource donation rates through thorough data collection. This should encompass surveys of potential donors, analysis of successful donation campaigns, and an understanding of regional variations in donation patterns. It is essential to examine not only individual motivations but also structural barriers, such as inconvenient donation points, limited operating hours, inadequate follow-up care for donors, and insufficient recognition mechanisms. Without this fundamental understanding, relevant interventions risk repeating past mistakes.

 Second, transitional mechanisms must acknowledge current realities while building capacity. Current research suggests that purely altruistic appeals may be insufficient in the current social context.^21^ China should experiment with non-monetary incentives validated, such as “priority points” for future medical use that are portable across provinces, or digital recognition badges that appeal to younger demographics. These local strategies align with successful global precedents, such as Brazil, which institutionalized non-monetary incentives like state-mandated donor leave to sustain engagement within the urban workforce. Leveraging big data to predict repeat donor behavior can also optimize recruitment precision.^22^

 Third, structural issues require structural solutions to address the geographic mismatch between medical facilities and donor populations. This necessitates the development of regional medical centers and a robust national blood allocation system. Similar to established organ sharing networks, a digitized blood inventory system could automate the redistribution of supplies from blood-rich provinces to major medical centers, effectively insulating individual hospitals from recruitment pressures, a centralized model that has helped countries like Namibia achieve self-sufficiency. Technology solutions should focus on reducing the “friction” of donation and curbing informal broker markets through real-time inventory transparency, mirroring India’s success in rebuilding public trust through visibility. Ultimately, bridging the policy-practice gap requires this synchronized approach: strengthening national redistribution infrastructure while formalizing incentives that recognize the donor’s social contribution.

## Conclusion

 China’s blood donation paradox ultimately reveals a fundamental truth about health policy and management. Effective health systems emerge not from policy declarations alone, but from systems where official rules and operational realities. The 2018 policy transition was undoubtedly a principled effort to meet global safety standards; however, as this analysis suggests, even the most well-intentioned mandates can falter when they outpace institutional capacity or encounter unforeseen disruptions like the COVID-19 pandemic. When policies ignore practical constraints, when prohibition occurs without ensuring alternatives, and when the gap between mandate and practice becomes routine, the result is a system that fails its most vulnerable populations, specifically those who lack the connections or resources to navigate informal channels.

 Addressing this disconnect requires moving beyond mere declaration toward genuine health system development. This involves building voluntary capacity based on evidence, ensuring equitable access through transparent mechanisms, and maintaining a pragmatic alignment between policy intentions and operational capabilities.^23^ While this study is constrained by its qualitative nature and the difficulty of quantifying clandestine markets, it nonetheless reveals a critical systemic mismatch. Furthermore, while we focus here on institutional and operational drivers, the evolving social values of core donor demographics represent a distinct sociological dimension that warrants future interdisciplinary inquiry. All individuals, regardless of social status or economic means, should have fair opportunity to access high-quality care they need.^24^

 China’s experience offers valuable lessons for health systems worldwide: sustainable change requires not just good policies but the infrastructure, evidence, and adaptive mechanisms necessary to implement them effectively. The true quality measure of any healthcare system lies not in the elegance of its policies or the ambition of its reforms, but in its capacity to translate intentions into accessible, equitable care for every patient who seeks healing.

## Disclosure of artificial intelligence (AI) use

 Not applicable.

## Ethical issues

 Not applicable.

## Conflicts of interest

 Authors declare that they have no conflicts of interest.
